# Tracking vegetation phenology across diverse biomes using Version 2.0 of the PhenoCam Dataset

**DOI:** 10.1038/s41597-019-0229-9

**Published:** 2019-10-22

**Authors:** Bijan Seyednasrollah, Adam M. Young, Koen Hufkens, Tom Milliman, Mark A. Friedl, Steve Frolking, Andrew D. Richardson

**Affiliations:** 1 Northern Arizona University, School of Informatics, Computing, and Cyber Systems, Flagstaff, AZ 86011 USA; 2 Northern Arizona University, Center for Ecosystem Science and Society, Flagstaff, AZ 86011 USA; 3 Harvard University, Department of Organismic and Evolutionary Biology, Cambridge, MA 02138 USA; 4 Faculty of Bioscience Engineering, Ghent University, Ghent, 9000 Belgium; 5 INRA, UMR ISPA, Villenave d’Ornon, France; 6 University of New Hampshire, Earth Systems Research Center, Durham, NH 03824 USA; 7 Boston University, Department of Earth and Environment, Boston, MA 02215 USA

**Keywords:** Phenology, Environmental impact, Ecosystem ecology, Ecosystem ecology

## Abstract

Monitoring vegetation phenology is critical for quantifying climate change impacts on ecosystems. We present an extensive dataset of 1783 site-years of phenological data derived from PhenoCam network imagery from 393 digital cameras, situated from tropics to tundra across a wide range of plant functional types, biomes, and climates. Most cameras are located in North America. Every half hour, cameras upload images to the PhenoCam server. Images are displayed in near-real time and provisional data products, including timeseries of the Green Chromatic Coordinate (Gcc), are made publicly available through the project web page (https://phenocam.sr.unh.edu/webcam/gallery/). Processing is conducted separately for each plant functional type in the camera field of view. The PhenoCam Dataset v2.0, described here, has been fully processed and curated, including outlier detection and expert inspection, to ensure high quality data. This dataset can be used to validate satellite data products, to evaluate predictions of land surface models, to interpret the seasonality of ecosystem-scale CO_2_ and H_2_O flux data, and to study climate change impacts on the terrestrial biosphere.

## Abstract (summary)

**Table:** 

Measurement(s)	plant phenological trait • plant greenness
Technology Type(s)	digital camera
Sample Characteristic - Organism	Embryophyta
Sample Characteristic - Environment	terrestrial biome
Sample Characteristic - Location	Earth (planet)

Machine-accessible metadata file describing the reported data: 10.6084/m9.figshare.9913694

## Background & Summary

Phenology is broadly defined as the timing of recurring of biological events^1^. Vegetation phenology exerts significant control over seasonal changes in ecosystem structure and function^2,3^. Key examples of this influence include the role of vegetation phenology in dictating the timing and magnitude of ecosystem carbon uptake^4,5^, as well as seasonal shifts in energy and water fluxes between the surface and the atmosphere^6–8^. Vegetation phenology is also sensitive to climate variability. In temperate and boreal forest ecosystems, phenology is predominantly driven by air temperature^9–11^, while in warm, arid grassland ecosystems, phenology responds strongly to the timing and magnitude of precipitation events^12,13^.

Standardized and publicly-available phenology datasets can provide a key source of information to aid scientists and land managers in documenting—and anticipating—the impacts of climate change on terrestrial ecosystems^14,15^. A range of vegetation phenology datasets are available, varying in spatial and temporal extent and resolution^16^. On-the-ground observations of individual organisms have been made by citizen science observers for decades, and contributed to databases such as the U.S. National Phenology Network^17^. At much broader spatial scales, satellite-based remote sensing platforms (e.g., Moderate Resolution Imaging Spectroradiometer (MODIS), Landsat, and Sentinel 2) provide time-series of land-surface greenness (e.g., normalized difference vegetation index), allowing for characterization of vegetation phenology across the earth’s entire surface^18,19^. However, landscape heterogeneity is unresolved under relatively course pixel resolutions provided by satellites sensors (e.g., ≈500 meters in MODIS), potentially confounding phenological signals^20,21^. Over the last decade, a complementary approach has been developed to monitor vegetation phenology: near-surface remote sensing using high-frequency digital repeat photography^16,22–25^. While both airborne and ground-based observations have their own strengths, limitations and uncertainties, this so-called “phenocam” approach can serve as a bridge across scales, providing continuous temporal coverage of phenological change at the organism-to-ecosystem level, with comparatively small uncertainties^26^.

Digital repeat photography offers an automated and cost-effective way to characterize temporal changes in vegetation^16^. Briefly, digital cameras, mounted overlooking the vegetation of interest, are used in time-lapse mode to record images throughout the day, from sunrise to sunset. Information about vegetation color—most commonly, “canopy greenness”—is extracted from the imagery, and used to quantify phenological changes. Specific phenophase transition dates, e.g. corresponding to the onset of spring green-up, can be identified from the seasonal trajectory of canopy greenness. Image analysis can be conducted for individual organisms or at the canopy scale. For more information, see refs^16,22,27,28^.

The PhenoCam network (http://phenocam.sr.unh.edu) was established in 2008. PhenoCam, which focuses on terrestrial ecosystems of North America, is one of several networks worldwide to leverage near-surface remote sensing for tracking of vegetation phenology. Similar networks include the European Phenology Camera Network (EuroPhen)^29^ and the Japanese Phenological Eyes Network (PEN)^30^. Phenocams are also being deployed as part of the NEON (National Ecological Observatory Network) and LTAR (Long Term Agricultural Research) networks in the USA, ICOS (Integrated Carbon Observation System) in Europe, TERN (Terrestrial Ecosystem Research Network) and the Australian Phenocam Network in Australia, and the e-Phenology project in Brazil^16,31–34^.

**Fig. 1:**
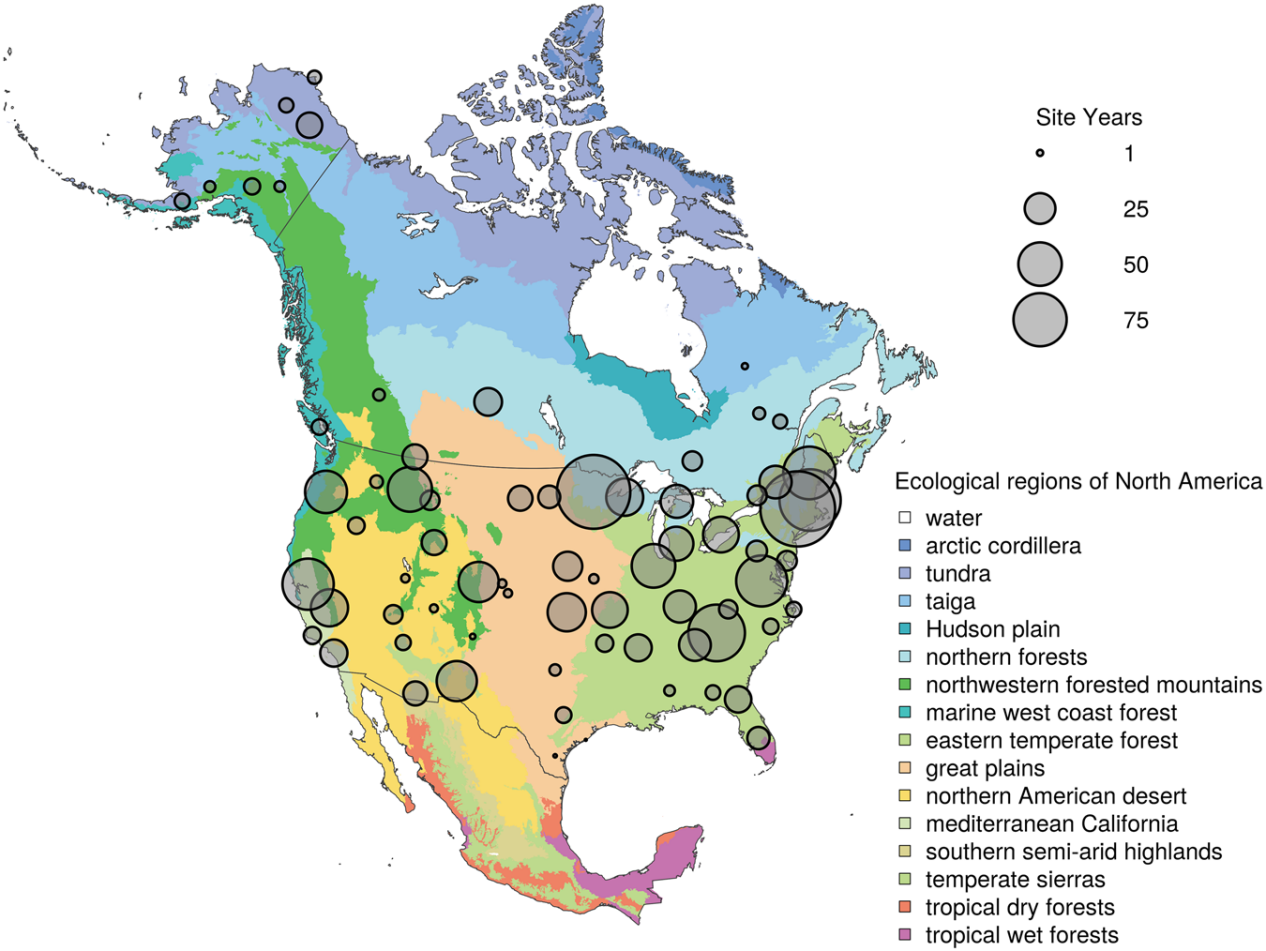
Spatial distribution of PhenoCam data across ecological regions of North America. Background map illustrates USA Environmental Protection Agency Level I Ecoregions^1,51^. Data counts have been aggregated to a spatial resolution of 4°, and the size of each circle corresponds to the number of site-years of data in the 4 × 4° grid cell. Sites in Hawaii, Puerto Rico, Central and South America, Europe, Asia and Africa (total of 88 site years) are not shown.

The previously-released PhenoCam Dataset v1.0^28^ is a curated and publicly available (CC0 Public Domain Dedication) data set that includes both digital camera imagery (10.3334/ORNLDAAC/1560)^35^ and data derived from that imagery (10.3334/ORNLDAAC/1511)^36^. This initial public release of data from PhenoCam included imagery through the end of 2015 from 130 cameras, comprising almost 750 site-years of data^28^. Here, we describe a major update—v2.0—to the PhenoCam Dataset, with two significant improvements. First, coverage has been substantially increased. The new dataset includes imagery through the end of 2018 from 393 cameras, comprising 1783 site-years of data (see Fig. 1). Second, data and imagery have been screened to exclude cameras programmed for “grey world” automatic white–balancing (AWB), as AWB can negatively impact the quality of the derived time series. In addition, we also present here an analysis of the representativeness of the current distribution of PhenoCam network cameras, to identify ecoregions and climate zones that are under-represented.

## Methods

Details on camera installation and configuration protocols, site classification, and image and data processing routines have been previously documented by Richardson, *et al*.^28^. Here we provide a brief summary.

Each PhenoCam camera is classified into one of three classes: Type I, Type II or Type III. Type I cameras (406 cameras) follow a standardized protocol, and site personnel are actively engaged as PhenoCam collaborators, e.g. providing camera maintenance and troubleshooting as required. For Type II cameras (70 cameras), there is some deviation from the standard protocol (e.g., non-standard camera brand or model), but site personnel are still actively engaged. For Type III cameras (52 cameras), there is some deviation from the standard protocol, and no active collaboration of personnel on-site.

All cameras in the PhenoCam network record three-layer JPEG images, from which we extract information about the mean intensity of each of the red, green and blue (RGB) color channels, calculated across a user-defined region of interest (ROI). The ROI corresponds to the vegetation under study. While there are a variety of ways in which this color information can be used^3,37^, the Green Chromatic Coordinate (*G*_CC_) is a commonly-used metric which has been applied successfully in many ecosystems^16,22^:

1 $${G}_{CC}=\frac{{G}_{DN}}{{R}_{DN}+{G}_{DN}+{B}_{DN}}$$

where *R*_*DN*_, *G*_*DN*_ and *B*_*DN*_ are the average red, green and blue digital numbers (a measure of intensity) across the ROI, respectively. Similarly, the red and blue chromatic coordinates are defined as normalized red and blue digital numbers. The red chromatic coordinate has been shown to be particularly well-suited to characterizing the timing of peak autumn colors in many deciduous forests^38^.

While a single image per day would be generally sufficient to document phenological changes in most ecosystems, it is typical for cameras in the PhenoCam network to upload an image every 15 or 30 minutes. This ensures high quality data by minimizing data discontinuity in cases of unfavorable weather (rain or snow), adverse illumination conditions (clouds or aerosols), or short-term power outages. Following previously-developed methods^22^, we use the sub-daily *G*_CC_ time-series (calculated for each image) to generate 1-day and 3-day “summary product” *G*_CC_ timeseries. The 1-day and 3-day time series were obtained from the 90th percentile of canopy greenness at 1-day and 3-day intervals, respectively^22^. The 1-day time series have finer temporal resolution, whereas the 3-day time series generally have less high-frequency noise. From the summary product time series, we then calculate phenological transition dates corresponding to the start of each “greenness rising” phenological phase, and the end of each “greenness falling” phenological phase. Uncertainties are quantified for all transition date estimates. In Richardson, *et al*.^28^, we erroneously indicated that a spline-based method was used to detect outliers in the greenness time-series data. The method we used was locally weighted scatterplot smoothing (i.e. LOESS).

## Data Records

The PhenoCam Dataset v2.0 consists of five different Data Records. Data Record 1 contains general metadata for each camera site, whereas Data Records 2 through 5 have been calculated for specific ROIs from each camera. For example, Data Record 2 contains the files used for image processing steps, i.e. ROI mask files, and information about the range of images over which these should be applied. Data Record 3 contains time-series of color-based statistics (e.g., chromatic coordinates), calculated for each image in the archive. Data Record 4 contains “summary product” 1-day and 3-day time-series for a variety of phenologically-relevant color-based metrics. Data Record 5 contains phenological transitional dates (i.e., phenophases) obtained from the 1-day and 3-day summary time-series. The data records are organized as follows for each camera site.

<sitename>

└─── data_record_1 (contains general metadata for each camera site)

- <sitename>_meta.json
- <sitename>_meta.txt
  └───data_record_2 (contains the ROI list files and image mask files used for image processing)
- <sitename>_ <veg_type> _ <ROI_ID_number> _roi.csv
- <sitename>_ <veg_type> _ <ROI_ID_number> _ <mask_index> .tif
  └─── data_record_3 (contains time series of ROI color statistics, calculated for each image in the archive, using data_record_2)
- <sitename>_ <veg_type> _ <ROI_ID_number> _roistats.csv
  └─── data_record_4 (contains time series of ROI color summary statistics, calculated for 1 and 3 day aggregation periods from data_record_3)
- <sitename>_ <veg_type> _ <ROI_ID_number> _1day.csv
- <sitename>_ <veg_type> _ <ROI_ID_number> _3day.csv
  └─── data_record_5 (contains phenological transition dates, calculated from data_record_4)
- <sitename>_ <veg_type> _ <ROI_ID_number> _1day_ transition_dates.csv
- <sitename>_ <veg_type> _ <ROI_ID_number> _3day_transition_dates.csv

Here, <sitename> is the name of each camera site, < veg_type > is a two-letter code defining the type of vegetation for which data have been processed (see Table 1), and < ROI_ID_number > is a unique identifier to distinguish between multiple ROIs of the same vegetation type for a given site. Together, these five data records are contained within Seyednasrollah^39^, and are derived from the imagery in Milliman^40^.

**Table 1:** Vegetation type abbreviations for ROIs (region of interests), and the corresponding number of site-years of data in the PhenoCam dataset described here (v2.0). For comparative purposes, the number of site-years of data in the previous dataset release (v1.0) is also presented. The absence of MX ROIs in the v2.0 data release is due to the fact that we have delineated separate ROIs for the plant functional types comprising the mixed stand (i.e., separation of EN and DN ROIs). Note that non-vegetated ROIs are not included in the v2.0 data release.

Abbreviation	Description	Site-years in Dataset v1.0	Site-years in Dataset v2.0
AG	agriculture	50	226
DB	deciduous broadleaf	392	643
DN	deciduous needleleaf	4	45
EB	evergreen broadleaf	2	28
EN	evergreen needleleaf	80	265
GR	grassland	121	279
MX	mixed vegetation (generally EN/DN, DB/EN, or DB/EB)	5	—
NV	non-vegetated	14	—
SH	shrubs	46	142
TN	tundra (includes sedges, lichens, mosses, etc.)	22	62
UN	understory	—	18
WL	wetland	11	64

The structure of the data records in the PhenoCam Dataset v2.0 is identical to that of the PhenoCam Dataset v1.0, and users of the Dataset v2.0 are directed to the user guide (https://daac.ornl.gov/VEGETATION/guides/PhenoCam_V2.html) and our previous data descriptor^28^ for an explanation of the headers and columns in each data record.

## Technical Validation

### Efforts to maintain quality control

The PhenoCam image archive and derived data products are regularly reviewed by the PhenoCam project team (the authors of this Data Descriptor) to maintain the quality of the dataset. This consists of visual inspection of the imagery for each site, and of the *G*_CC_ time series data derived from the imagery and displayed in near-real time on the project web page. Imagery from each PhenoCam is also checked for field of view shifts, interruptions to data continuity, and camera misconfigurations. Should any issues be identified, site contacts are notified by email, and asked to investigate and implement corrective measures if possible. In the case of field of view shifts, ROI masks are adjusted or redrawn as needed, and the imagery is reprocessed to ensure that the effects of field-of-view shifts are minimized^28^.

We previously presented extensive documentation of the steps taken to ensure that data derived from PhenoCam imagery are of the highest quality^28^. This documentation included analyses of independent data sources. For example, we demonstrated excellent agreement between both direct observations of vegetation phenology and phenocam-derived metrics, as well as between vegetation indices derived from radiometrically-calibrated measurements of surface reflectance (e.g. with narrow-band, tower-mounted sensors) and phenocam-derived vegetation indices such as *G*_CC_.

Here we report more recent efforts (1) to identify and exclude data and imagery from cameras erroneously set to auto white balance; and (2) to assess the spatial representativeness of the PhenoCam network in the context of the biological and climatological variability of ecosystems across North America.

### Auto white balancing

In digital photography and image processing, “white balancing” is the practice of adjusting digital numbers for each color channel in order to produce a neutral image (i.e. the “grey world” model) for given red (R), green (G), and blue (B) values. This can be useful under varying conditions of illumination, with the intended effect of rendering white and grey tones correctly^41^. Therefore, most digital cameras (particularly consumer-grade “point-and-shoot” cameras) perform some sort of automatic white balance (AWB) “correction.” The outcome may appear more pleasant to the human eye, as the adjusted colors correspond more closely to human perception of the scene. While this could compensate for changing illumination conditions in PhenoCam images, the AWB correction often results in color inconsistency as the scene changes, for example as leaves emerge in spring, or as the sky color changes from grey to blue. As a result, the chromatic coordinates obtained from AWB images may not be suitable for quantifying vegetation phenology: the data may be noisy^42^, or even wrong.

**Fig. 2:**
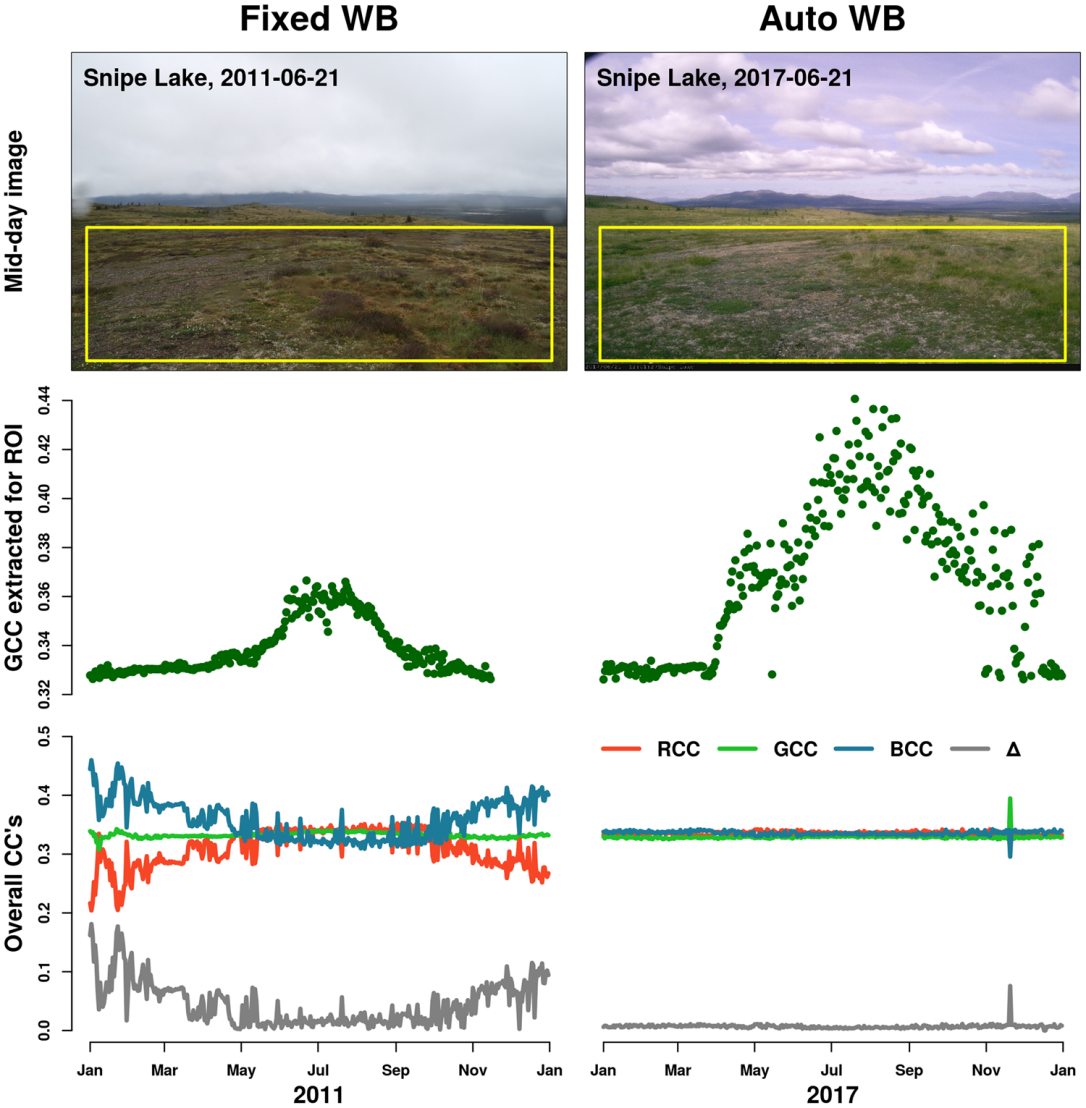
Qualitative and quantitative effects of Auto-White Balance (AWB) on phenological data. The left and right panels show when AWB was off and on at the Snipe Lake PhenoCam site (60.6°N, 154.3°W, tundra), respectively: (**a**) sample images taken on the same day (summer solstice) of the year in 2011 and 2017, (**b**) full-year green chromatic coordinates extracted from an ROI representative of vegetation greenness, and (**c)** the red, green and blue chromatic coordinates (*R*_CC_, G_CC_ and *B*_CC_) and the deviation from grey (Δ) extracted from the whole image.

Figure 2 shows an example of how AWB can affect both the digital images themselves, and the extracted *G*_CC_ time-series, using imagery from the Snipe Lake PhenoCam site. In 2011, the Snipe Lake camera was set to fixed white balance, whereas in 2017 the camera was set to AWB. The purple cast of the sky, visible in the 2017 image (Fig. 2a), is the direct result of AWB compensating for the greenness of the foreground vegetation. The white balance setting also influences the derived *G*_CC_ time series, which is not only noisier for the 2017 (AWB) imagery than the 2011 (non-AWB) imagery, but also leads to mischaracterization of vegetation seasonality: the rise in *G*_CC_ in March 2017 is due to snowmelt, rather than vegetation greening up. These artifacts are the product of AWB counteracting changes in scene and illumination by adjusting the color sensitivity of the imaging sensor so that across the entire image, the mean color is grey, and each of the red, green, and blue chromatic coordinates is approximately equal (0.33). The lack of any seasonal patterns in the whole-image chromatic coordinates in 2017 contrasts sharply with the seasonality evident in 2011 (Fig. 2c), and provides confirmation that the camera is configured for AWB using the “grey world” method.

The PhenoCam configuration protocol specifies that all cameras should be set to fixed white balance. On the StarDot cameras that are used at Type I sites, this involves setting the color balance to “manual” and adjusting the color skew values to custom settings (R = 385, G = 256, B = 330). This configuration is implemented by the PhenoCam Installation Tool (PIT; http://khufkens.github.io/phenocam-installation-tool/).

Because of the negative impact of AWB on PhenoCam imagery and data products, we have implemented several procedures to identify whether a given camera may be recording imagery using the “grey world” AWB model. For standard PhenoCam cameras (Type I), configured using the PIT, the metadata text file associated with each image reports whether the camera is on manual (i.e. fixed) color balance (“balance = 0”) or AWB (“balance = 1”), and these files are scanned regularly to identify cameras which have been erroneously set to AWB.

For non-standard cameras (Type II and Type III), we have developed an *ad hoc* method to identify potential AWB cameras. Briefly, we define the deviation from grey, Δ, as in Eq. 2:

2 $$\Delta ={\left[{\left(\frac{\bar{{R}_{DN}}}{\bar{{R}_{DN}}+\bar{{G}_{DN}}+\bar{{B}_{DN}}}-\frac{1}{3}\right)}^{2}+{\left(\frac{\bar{{G}_{DN}}}{\bar{{R}_{DN}}+\bar{{G}_{DN}}+\bar{{B}_{DN}}}-\frac{1}{3}\right)}^{2}+{\left(\frac{\bar{{B}_{DN}}}{\bar{{R}_{DN}}+\bar{{G}_{DN}}+\bar{{B}_{DN}}}-\frac{1}{3}\right)}^{2}\right]}^{1/2}$$

Here $\bar{{R}_{DN}}$, $\bar{{G}_{DN}}$, and $\bar{{B}_{DN}}$ are the average red, green and blue (respectively) digital numbers, calculated across the *entire* image. As shown for the Snipe Lake imagery in Fig. 2c, Δ tends to be very close to zero when cameras are on AWB. We identify images with Δ < 0.02 for more than 30 consecutive days as “AWB suspects” and conduct further investigation. In some cases, imagery from cameras not on AWB has low Δ because the image is dominated by neutral tones—when the ground is snow-covered, for example. But, if further investigation leads to the conclusion that the camera was likely on AWB, that imagery has been excluded from this dataset. Data from roughly a dozen camera sites has been excluded because of concerns about poor-quality data resulting from AWB.

We note that this approach is imperfect; on some cameras, for example, only the brightest pixels are used to determine the white balance, and in this case our method would not necessarily work. We are working on the development of more general methods to detect AWB imagery.

### Comparison of Transition Dates between the PhenoCam Dataset v1.0 and v2.0

**Fig. 3:**
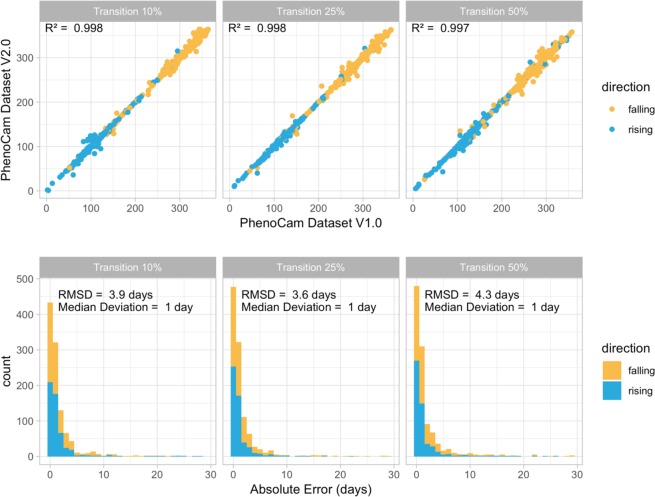
Comparison of transition dates between the PhenoCam dataset v1.0 and v2.0. The intercomparison showed a strong agreement between the two versions.

As discussed above, the processing steps and data quality of the PhenoCam dataset have been improved from v1.0 to v2.0 but the new release of the dataset does not invalidate the previous version. We showed this by comparing 535 rising and 577 falling seasonal cycles that were common between the two versions (Fig. 3). We compared the rising and the falling 10%, 25% and 50% transition dates between the two datasets, based on the 3-day 90^th^ percentile GCC timeseries. The intercomparison showed a strong agreement (R^2^ > 0.99 for all the 10%, 25 and 50% transition dates) between v1.0 and v2.0. Median absolute error was only 1 day for all the 10%, 25 and 50% transition dates. Root mean square deviation (RMSD) was 3.9, 3.6 and 4.3 days for the 10%, 25 and 50% transition dates, respectively. We identified less than 1% of the transition dates were the difference was more than 20 days. The small discrepancies between the two datasets may be caused by several factors, including updated masks, and newly available data since the last release. For example, the revised masks for howland1 EN 2000 resulted in a shift of 22 days in transition 50% of year 2008, because the old mask had contaminated with a deciduous signal. In another instance, the 2015 end-of-season transition dates for turkeypointenf39 EN 1000 shifted more than two weeks, caused by the properly constrained winter baseline with the additional data for 2016 onward.

### Representativeness of the PhenoCam Network

Cameras in the PhenoCam network are widely distributed across North America, and the over-arching objective of the network is to sample the diversity of ecosystems and climate zones across the continent. Cameras located at sites with a range of different vegetation types (Table 1), including agriculture (226 site years), deciduous broadleaf (643 site years), deciduous needleleaf (45 site years), evergreen broadleaf (28 site years), evergreen needleleaf (265 site years), grassland (279 site years), shrub (142 site years), tundra (62 site years), understory (18 site years), and wetland (64 site years).

**Fig. 4:**
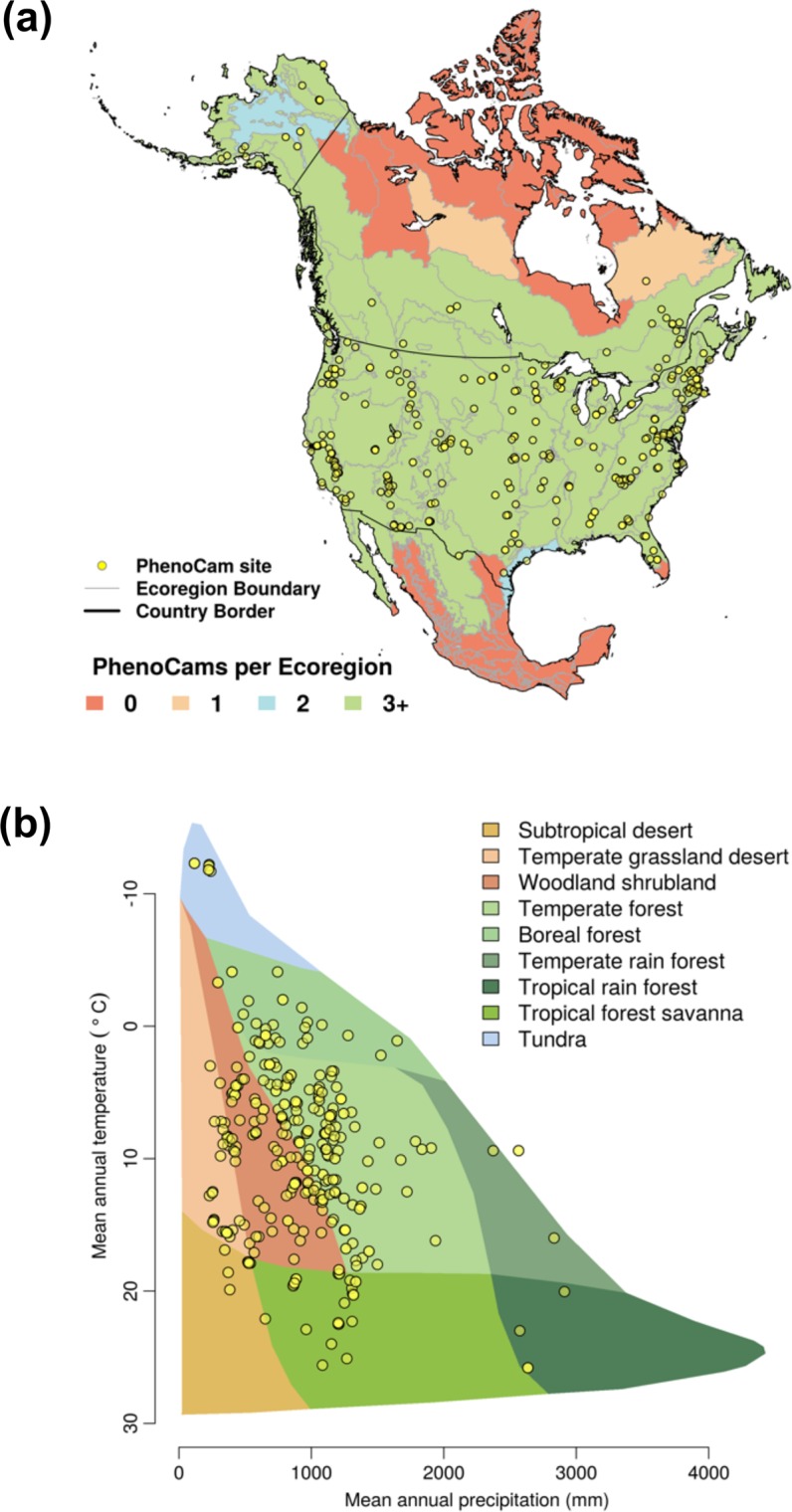
Representativeness of the PhenoCam cameras. (**a**) Distribution of PhenoCam sites across North America, with Level II Ecoregions colored by the number of PhenoCams per region; and (**b**) Distribution of PhenoCams across the global vegetation biomes defined by the Whittaker classification. Ecoregions boundaries are obtained from Ecoregion Level II of United States Environmental Protection Agency (https://www.epa.gov/eco-research/ecoregions-north-america).

To more comprehensively investigate the degree to which PhenoCams are distributed across the biotic and abiotic variability of ecosystems in North America, we use two approaches. First, using the Level II Ecoregion classification of North America (https://www.epa.gov/eco-research/ecoregions-north-america), we identified those areas where coverage is lowest. From about 30°N to 55°N, virtually every Level II ecoregion has at least three (and in many cases substantially more) PhenoCams in it (Fig. 4a). Ecoregions of interior Alaska, central and far northern Canada (much of this area is sparsely vegetated^43^ and also relatively inaccessible), the gulf coast of Texas, the southern tip of Florida, and most of Mexico emerge as poorly-represented in this analysis. These are areas that should be targeted for further expansion of the network.

Second, using the Whittaker Biome Classification^44,45^, we examined the distribution of PhenoCam sites across global climate-space (Fig. 4b). Mean annual temperature at PhenoCam sites spans almost 40 °C, ranging from −12.3 °C to 25.8 °C, while mean annual precipitation varies 30-fold, from just over 100 mm to over 3000 mm. Among the biomes corresponding to this climatic variability, boreal forest, temperate forest, temperate grassland desert, temperate rain forest, tropical forest savanna, and woodland/shrubland biomes are generally well-represented by the current distribution of PhenoCam network sites. However, the climate representativeness of the network would benefit from the installation of more cameras in subtropical desert, tundra, and tropical rain forest biomes.

## Usage Notes

The curated PhenoCam Dataset v2.0 is permanently and publicly available through the Oak Ridge National Lab (ORNL) DAAC (Distributed Active Archive Center for Biogeochemical Dynamics) data repository (10.3334/ORNLDAAC/1674). We have also developed an interface (http://explore.phenocam.us/) to facilitate data exploration and visualization, from which the user can also download data on a site-by-site basis. All imagery and data (updated in near-real time, and including data from sites where the data record is shorter than six months, or the data are not considered to be high enough quality, for inclusion in a curated data release) are also available through the project web page (http://phenocam.sr.unh.edu).

## Software Applications

The PhenoCam team has developed several software application and packages to facilitate extraction and processing of data from PhenoCam imagery. Code for each of these tools is made available on an open-source basis, for reuse and development by the community.

### xROI

*xROI* is an open-source *R* package to extract time-series data from large sets of digital images. With a graphical user interface, *xROI* provides functionality to delineate ROIs, to detect data discontinuities (FOV shifts, clouds, etc.), and to derive high-quality color-based statistics (digital numbers, chromatic coordinates) from stacks of images. The *xROI* software can be used to extract data from PhenoCam imagery for custom ROIs, or from imagery not included in the PhenoCam archive. While the package is available from the *R* Comprehensive Archive Network (CRAN), the source code (https://github.com/bnasr/xROI) is open under the GNU Affero General Public License (AGPLv3) and can be downloaded from refs^46,47^. For more details see Seyednasrollah *et al*.^48^.

### Hazer

**Fig. 5:**
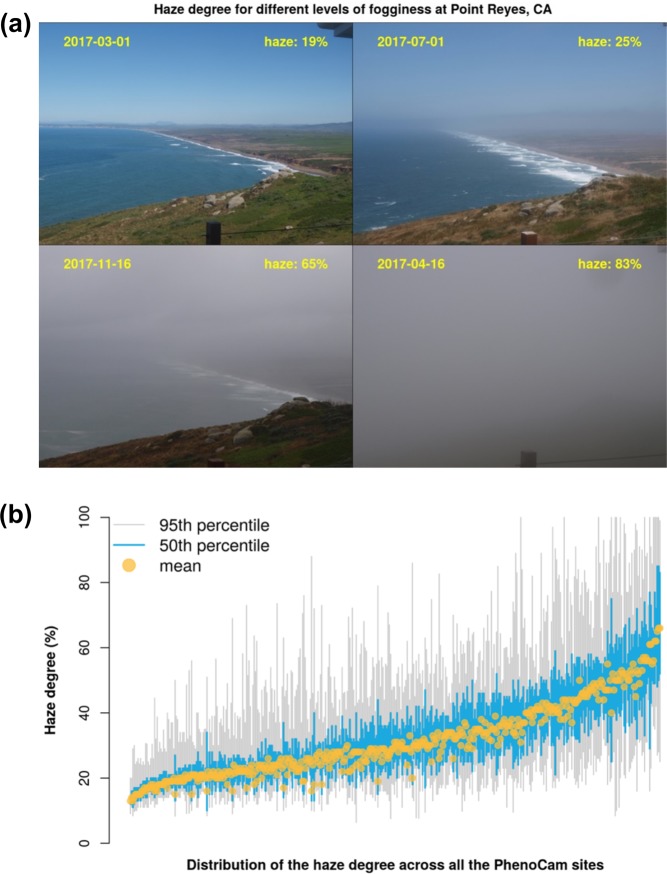
Haze degree estimated by hazer R package. (**a**) Haze degree values for different fogginess at the Point Reyes PhenoCam site located at 123.02°W and 37.99°N, and (**b**) distribution of haze degree across all PhenoCam sites (including sites not included in the dataset). On panel b, x-axis indicate PhenoCam sites sorted by their median haze degree values.

*hazer* is an open-source *R* package for detecting foggy or hazy images. Haze statistics are calculated from the frequency distribution of RGB digital numbers across the image. The *getHazeFactor* function returns the “haze degree” value, varying from 0 to 100%. High haze degree values indicate high probabilities of haze or fogginess (Fig. 5a). We consider images with haze degree > 40% to be foggy or hazy; the distribution of the haze degree value across all PhenoCam sites is shown in Fig. 5b. While the haze degree values are used for quality check, the hazy images are not excluded from the data release v2.0. The package also presents additional functionalities to obtain brightness, darkness and contrast matrices for an image. *Hazer* is open source under the GNU Affero General Public License (AGPL-v3)^49^.

### Phenocamr

The *phenocamr R* package facilitates the retrieval and post-processing of PhenoCam time series^50^. The post-processing of PhenoCam data includes outlier removal and the generation of data products, in particular the phenological transition dates as included in this dataset. The package is available from the *R* Comprehensive Archive Network (CRAN) while the source code is open under the GNU Affero General Public License v3.0 and available from https://github.com/khufkens/phenocamr.
