# Publisher Correction: Tracking vegetation phenology across diverse biomes using Version 2.0 of the PhenoCam Dataset

**DOI:** 10.1038/s41597-019-0270-8

**Published:** 2019-11-01

**Authors:** Bijan Seyednasrollah, Adam M. Young, Koen Hufkens, Tom Milliman, Mark A. Friedl, Steve Frolking, Andrew D. Richardson

**Affiliations:** 1 Northern Arizona University, School of Informatics, Computing, and Cyber Systems, Flagstaff, AZ 86011 USA; 2 Northern Arizona University, Center for Ecosystem Science and Society, Flagstaff, AZ 86011 USA; 3 Harvard University, Department of Organismic and Evolutionary Biology, Cambridge, MA 02138 USA; 4 Faculty of Bioscience Engineering, Ghent University, Ghent, 9000 Belgium; 5 IN RA, UMR ISPA, Villenave d’Ornon, France; 6 University of New Hampshire, Earth Systems Research Center, Durham, NH 03824 USA; 7 Boston University, Department of Earth and Environment, Boston, MA 02215 USA

Correction to: *Scientific Data* 10.1038/s41597-019-0229-9, published online 22 October 2019

In the original version of this Data Descriptor, the URL provided to access the accompanying machine-accessible metadata file was incorrect. This has now been corrected.

